# Optimization of the Load of Transition Metal Oxides (Fe_2_O_3_, Co_3_O_4_, NiO and/or PdO) onto CeO_2_ Nanoparticles in Catalytic Steam Decomposition of *n*-C_7_ Asphaltenes at Low Temperatures

**DOI:** 10.3390/nano9030401

**Published:** 2019-03-09

**Authors:** Oscar E. Medina, Jaime Gallego, Daniela Arias-Madrid, Farid B. Cortés, Camilo A. Franco

**Affiliations:** 1 Grupo de Investigación en Fenómenos de Superficie—Michael Polanyi, Departamento de Procesos y Energía, Facultad de Minas, Universidad Nacional de Colombia, Sede Medellín, 050034 Medellín, Colombia; oemedinae@unal.edu.co (O.E.M.); daariasma@unal.edu.co (D.A.-M.); 2 Química de Recursos Energéticos y Medio Ambiente, Instituto de Química, Universidad de Antioquia UdeA, Calle 70 No. 52-21, 050010 Medellín, Colombia; andres.gallego@udea.edu.co

**Keywords:** adsorption, asphaltene, catalytic steam gasification, ceria, nanoparticles, thermal EOR, transition elements

## Abstract

The main objective of this work is the catalyst optimization of Fe_2_O_3_-, Co_3_O_4_-, NiO- and/or PdO- (transition element oxides—TEO) functionalized CeO_2_ nanoparticles to maximize the conversion of asphaltenes under isothermal conditions at low temperatures (<250 °C) during steam injection processes. Adsorption isotherms and the subsequent steam decomposition process of asphaltenes for evaluating the catalysis were performed through batch adsorption experiments and thermogravimetric analyses coupled to Fourier-transform infrared spectroscopy (FTIR), respectively. The adsorption isotherms and the catalytic behavior were described by the solid-liquid equilibrium (SLE) model and isothermal model, respectively. Initially, three pairs of metal oxide combinations at a mass fraction of 1% of loading of CeNi1Pd1, CeCo1Pd1, and CeFe1Pd1 nanoparticles were evaluated based on the adsorption and catalytic activity, showing better results for the CeNi1Pd1 due to the Lewis acidity changes. Posteriorly, a simplex-centroid mixture design of experiments (SCMD) of three components was employed to optimize the metal oxides concentration (Ni and Pd) onto the CeO_2_ surface by varying the oxides concentration for mass fractions from 0.0% to 2.0% to maximize the asphaltene conversion at low temperatures. Results showed that by incorporating mono-elemental and bi-elemental oxides onto CeO_2_ nanoparticles, both adsorption and isothermal conversion of asphaltenes decrease in the order CeNi1Pd1 > CePd2 > CeNi0.66Pd0.66 > CeNi2 > CePd1 > CeNi1 > CeO_2_. It is worth mentioning that bi-elemental nanoparticles reduced the gasification temperature of asphaltenes in a larger degree than mono-elemental nanoparticles at a fixed amount of adsorbed asphaltenes of 0.02 mg·m^−2^, confirming the synergistic effects between Pd and Fe, Co, and Ni. Further, optimized nanoparticles (CeNi0.89Pd1.1) have the best performance by obtaining 100% asphaltenes conversion in less than 90 min at 220 °C while reducing 80% the activation energy.

## 1. Introduction

The demand for conventional crude oils increases every day worldwide, and it is expected that in the future these reserves will decrease substantially [1]. Consequently, the energy industries have shown great attention in recent years to the use of alternative techniques to supply the current energy consumption [2]. The use of fossil fuels such as heavy crude oil (HO) and extra heavy crude oil (EHO) has become an important source for the alternative energy supply [3]. This type of crude oil has a high content of heavy hydrocarbons, such as asphaltenes, that drastically increase the viscosity of crude oil and reduce the American Petroleum Institute (API) gravity [4]. Thermal methods are used with the aim of viscosity reduction for improving the mobility ratio, productivity, and recovery of this type of crude oil [5,6,7]. However, some of these methods such as steam injection processes do not generate changes in the quality of crude oil. This is due to the low temperatures of operation that do not allow chemical reactions such as aquathermolysis, steam reforming, water-gas shift, and braking of C-S bonds, among others, that could lead to the decomposition of high molecular weight compounds, and hence, a permanent upgrade of the physicochemical properties of the crude oil [8,9,10,11]. Generally, the temperatures employed in steam injection processes do not exceed 240 °C [12], while the decomposition temperature of asphaltenes in the presence of steam occurs around 450–550 °C [13,14,15].

The application of nanoscale technologies has recently been used as an alternative for improving the techniques above and enhance the oil recovery [16,17,18,19]. Several authors [20,21,22,23] have shown that highly adsorbent nanomaterials can capture the asphaltene molecules of crude oil due to their high affinity [24]. Posteriorly, these nanoparticles can be used as catalysts in processes such as steam injection [25], pyrolysis [26], and in-situ combustion [27], looking for the catalytic decomposition that leads to the improvement of the properties of the HO and EHO. There are two principal lines of work within the studies reported in the literature focused on the catalytic upgrading of HO and EHO through nanotechnology. The first one employs metal oxides nanoparticles, while the second uses functionalized nanoparticulated supports with metal oxides (composites materials or supporting hygroscopic salts materials—SHS). Within the first branch, many types of nanoparticles such as NiO, SiO_2_, TiO_2_, WO_3_, MgO, CaCO_3_, ZrO_2_, Al_2_O_3_, Fe_2_O_3_, and CO_3_O_4_, among others, have been evaluated with the final purpose of reducing the decomposition temperature and activation energy of the asphaltenes, calculated under isothermal [28] or iso-conversional models [15]. Metal oxide nanoparticles can lead to the reduction of the asphaltene decomposition temperature up to 300 °C, which is still a relatively high temperature for a steam injection process in oilfield [15,22,29,30,31,32]. In the case of functionalized materials such as nickel and palladium nanocrystals over nanoparticulated supports of alumina, silica, or TiO_2_, the asphaltene decomposition is achieved at low temperatures between 200 °C and 240 °C [32,33,34,35,36]. However, these studies with functionalized materials have not been conducted under isothermal conditions, which can be more representative of the steam injection processes under reservoir conditions. In the case of asphaltene decomposition under isothermal conditions, Nassar et al. [28] evaluated three metal oxides nanoparticles of Fe_2_O_3_, Co_3_O_4_, and NiO at 300 °C under an air atmosphere, being the NiO is the one with the best performance, achieving 100% of asphaltene conversion in less than 170 min. Cardona et al. [37,38] evaluated the catalytic steam decomposition of asphaltenes using a composite material of NiO and PdO nanocrystals over alumina support under isothermal conditions at 220 °C, which is a better approximation to steam injection processes in the industry. Under these conditions, a 90% of asphaltene decomposition was achieved in less than 150 min, and its effect on the upgrading of the physicochemical properties of an EHO was corroborated through displacement tests of steam injection [37].

Cerium dioxide (IV) has been evaluated in several catalytic studies due to its ability to absorb and release oxygen through the Ce^3+^/Ce^4+^ redox cycle [39,40]. Therefore, this lanthanide material may promote the aforementioned reactions by means of (a) the effect of the interactions between transition elements oxides (TEO) and ceria (CeO_2_) [41,42,43]; (b) the activity of the redox pair Ce^3+^/Ce^4+^; and (c) the hydrogen production [44,45,46]. Recently, it was demonstrated that Ni as a disperse phase linked to cerium as active support in the framework of Zeolite Socony Mobil-Five (MFI) presents a synergistic effect in the water-gas shift (WGS) reaction, accelerating its production at low temperatures of around 230 °C [47]. However, to the best of our knowledge, there are no studies reported in the scientific literature that evaluate and optimize the concentration of metals oxides on the CeO_2_ nanoparticles for the adsorption/cracking processes of *n*-C_7_ asphaltenes under isothermal conditions focused on thermal EOR processes of steam injection. Therefore, the main objective of this study was to find the best combination of transition elements oxides (Pd, Ni, Co, and Fe) and their optimum concentration in the surface of a CeO_2_ nanoparticulated support that allow for the improvement of the conversion of *n*-C_7_ asphaltene during steam injection processes at low temperatures (<240 °C) under isothermal conditions, which can further lead to the HO and EHO upgrading. Hence, three TEOs are combined with a noble element oxide in the couples Fe–Pd, Co–Pd, Ni–Pd, and are functionalized over CeO_2_ nanoparticles for further evaluation through batch adsorption experiments and thermogravimetric analyses. Finally, through a simplex-centroid mixture design (SCMD) of experiments, the optimum concentration of the best metallic oxide pair is determined to maximize the *n*-C_7_ asphaltenes conversion under isothermal conditions at 220 °C. Therefore, this work will allow for a better understanding of the catalytic effect of transition metals, noble metals, and lanthanides for the decomposition of asphaltenes in steam injection processes, as well as the variables that can affect this process.

## 2. Materials and Methods

### 2.1. Materials

The extraction of the asphaltenes was performed by isolation with *n*-heptane (99%, Sigma-Aldrich, St. Louis, MO, USA) [48,49] from a Colombian extra heavy crude oil (EHO) of 6.4° API, viscosity of 3.1 × 10^6^ cP at 25 °C, and approximate mass fractions of saturates, aromatics, resins and asphaltenes (SARA) of 13.0%, 16.9%, 49.9%, and 20.2%, respectively. The *n*-C_7_ asphaltenes were characterized by elemental analysis using an elemental analyzer Flash EA 1112 (Thermo Finnigan, Milan, Italy), obtaining mass fractions of C, H, O, N, and S of 81.7%, 7.8%, 3.6%, 0.3% and 6.6%, and a H/C ratio of 1.15, which is in accordance with the values reported in literature [50]. Ceria (CeO_2_) nanoparticles were purchased from Nanostructured & Amorphous Materials (Houston, TX, USA). Salt precursors of NiCl_2_∙6H_2_O, CoCl_2_ ∙6H_2_O, FeCl_3_∙6H_2_O, Pd(NO_3_)_2_∙2H_2_O (Merck KGaA, Darmstadt, Germany), and distilled water were used for the functionalization of ceria nanoparticles.

### 2.2. Methods

#### 2.2.1. Functionalization of CeO_2_ Nanoparticles with NiO, Fe_2_O_3_, Co_3_O_4_, and PdO

For obtaining the optimum concentration of the best functionalizing couple on the cerium oxide nanoparticles, the following systems were considered: (i) initially three pairs of nanoparticles were evaluated with different TEO at a fixed concentration of a mass fraction of 1% over the ceria surface (CeNi1Pd1, CeCo1Pd1 and CeFe1Pd1) to choose the best TEO couple, and posteriorly (ii) a three-component simplex centroid mixture design (SCMD) of experiments was carried out for different TEO dosages up to mass fractions of 2%. The support of CeO_2_ nanoparticles was previously dried at 120 °C for 2 h to remove any humidity. For the functionalization, aqueous solutions of iron chloride, nickel chloride, cobalt chloride, and/or palladium nitrate are employed using the incipient wetness technique as reported in previous studies [23,37,51]. Then, the obtained composite nanoparticles were dried at 120 °C for 6 h, and finally, the solid obtained was calcined at 450 °C for 6 h [15]. The nomenclature, as well as the mass fraction and mole fraction of the synthesized samples according to the design of experiments, are shown in Table 1.

It is important to mention that Ni, Pd, Co, and Fe precursors become oxides after calcination. Existence of Co_3_O_4_ was confirmed through Raman spectroscopy as reported in Figure S1 of the Supplementary Materials Document.

The first part of this manuscript consists of three composite bimetallic materials based on a load of a fixed mass fraction of 1.0% of nickel and palladium oxides supported on the CeO_2_ nanoparticles (CeNi1Pd1), 1.0% of iron and palladium oxides (CeFe1Pd1), and 1.0% of cobalt and palladium oxides (CeCo1Pd1). In the second part, seven samples were prepared with the salt precursors according to the SCMD, varying the mass fraction between 0.0% and 2.0% of nickel and palladium oxides according to Figure S2 of the Supplementary Materials Document. The functionalized nanoparticles were labeled by the chemical symbol of the support (Ce), followed by the symbol of the transition element and its percentage. For instance, nanoparticles with loads of a mass fraction of 1.0% of NiO and PdO, are labeled as CeNi1Pd1.

#### 2.2.2. Characterization of the Nanoparticles

The size and morphology of the composite nanoparticles and the support were characterized by high-resolution transmission electron microscopy (HR-TEM) using a Tecnai G2 F20 microscope (FEI, Hillsboro, OR, USA) and dynamic light scattering (DLS) measurements using a nanoplus-3 from Micromeritics (Norcross, GA, USA). Metal dispersion and the average diameter of the metals in the catalyst support were found by pulse chemisorption, using H_2_ titration with a Chembet 3000 (Quantachrome Instruments, Boynton Beach, FL, USA), where approximately 100 mg of sample was subjected to a U-shaped quartz tube dried at 200 °C for 1 h. Then the catalysts were subjected to 600 °C for 1 h in a volumetric fraction of 10% of H_2_ in Ar at 80 mL·min^−1^ and purged with flowing Ar for 1 h until the samples reach room temperature. Hydrogen pulses continued until no additional uptake of H_2_ was observed. The surface area (S_BET_) was measured using N_2_ physisorption at −196 °C using an Aurosorb-1 Quantacrome (USA) following the method proposed by Brunauer-Emmett-Teller [40].

#### 2.2.3. Equilibrium Adsorption Isotherms

The model solutions for the batch adsorption experiments were prepared by dissolving a desired amount of the obtained asphaltenes in toluene. The procedure started with a stock solution containing 1500 mg·L^−1^ of *n*-C_7_ asphaltenes diluted at different concentrations. The initial concentration of *n*-C_7_ asphaltene solutions varied from 100 mg·L^−1^ to 1500 mg·L^−1^. A fixed amount of nanoparticles was added in a 1:10 ratio of solution volume to dry mass of nanoparticles. Subsequently, the mixtures were stirred at 200 rpm and allowed to equilibrate for 24 h, time needed to ensure the adsorption equilibrium [24]. Nanoparticles with asphaltenes adsorbed were separated by centrifugation at 5000 rpm for 45 min and dried in a vacuum oven at 60 °C for 24 h. Changes in the concentration of asphaltenes in solution after adsorption were determined by UV-vis spectrophotometry using a Genesys 10S spectrophotometer (Thermo Scientific, Waltham, MA, USA). The amount of adsorbed asphaltenes q (mg∙m^−2^) was determined by mass balance:

(1) $$q=\frac{C_{o}-C_{E}}{A}V$$

where $C_{E}$ (mg·L^−1^) is the equilibrium concentration of asphaltenes in the supernatant, $C_{o}$ (mg·L^−1^) is the initial concentration of *n*-C_7_ asphaltenes in solution, A (m^2^·g^−1^) is the dry surface area of nanoparticles, and V (L) is the solution volume.

#### 2.2.4. Thermogravimetric Analysis of Asphaltenes

Catalytic steam gasification of adsorbed *n*-C_7_ asphaltenes over the nanoparticles was carried out using a thermogravimetric analyzer Q50 (TA Instruments, Inc., New Castel, DE, USA) coupled to an IR-Affinity-1 FTIR device (Shimadzu, Kyoto, Japan) with a gas cell to analyze produced gases during the decomposition process. For gasification experiments, N_2_ flow was fixed at 100 mL·min^−1^ and at the same time H_2_O_(g)_ was introduced to the system at a flow rate of 6.30 mL·min^−1^ using a gas saturator filled with distilled water at a fixed temperature controlled by a thermostatic bath. This flow rate allows the steam to be present above the sample in excess [15].

The samples were subjected to two procedures under non-isothermal and isothermal conditions. Under non-isothermal conditions, the samples were heated from 100 °C to 600 °C at a heating rate of 20 °C·min^−1^. After the gasification process, oxidation under an air flow from 100 °C to 600 °C at 20 °C·min^−1^ was conducted to estimate the coke yield. Under isothermal conditions, the samples were heated at 230 °C, 240 °C, and 250 °C for 300 min to obtain the highest conversion of asphaltenes possible. In the case of *n*-C_7_ asphaltenes in the absence of nanoparticles, the temperatures for isothermal decomposition were established at 360 °C, 370 °C, and 380 °C due to the refractory nature of this compound. The selected nanomaterials for thermogravimetric analyses (TGA) experiments had the same loading of *n*-C_7_ asphaltenes per unit surface area of 0.2 mg·m^−2^. However, for the best system regarding catalytic activity, the effect of asphaltenes loading was evaluated for asphaltenes loadings of 0.02 mg·m^−2^, 0.05 mg·m^−2^ and 0.2 mg·m^−2^. To avoid diffusion limitations, the sample mass in the analyzer was kept low (~5 mg) [52].

The gases produced by the asphaltene decomposition process were analyzed via Fourier transform infrared spectroscopy (FTIR), which operated in transmission mode at a resolution of 2 cm^−1^ in the range of 4000–100 cm^−1^ with ten scans per minute. The gases that were produced by the cracking of asphaltenes were CH_4_, CO_2_, CO, NO_X_ and light hydrocarbons and their analysis was carried out considering their characteristic intensities of the absorption bands for each gas, which are 3016 cm^−1^, 2349 cm^−1^, 2149 cm^−1^, 1615 cm^−1^, and 2750 cm^−1^, respectively [16]. Each run was repeated at least twice to confirm the reproducibility of the experiment. Finally, the catalytic activity of the samples from the SCMD was evaluated under isothermal conditions of 220 °C and for an asphaltene load of 0.02 mg·m^−2^.

## 3. Modeling

### 3.1. Simplex-Centroid Mixture Design

A design of experiments was developed to find the optimal mixture of NiO or PdO and ceria nanoparticles for maximizing the conversion of the asphaltenes. A simplex-centroid mixture design (SCMD) was run using the STATGRAPHICS Centurion XVI software (StartPoint Technologies Inc., Addison, TX, USA) varying the mass fraction of palladium, cerium, and nickel. The SCMD is used to predict the response of a selected parameter according to the variability of a controlled variable. The proportion of each component must satisfy the following restriction [53]:

(2) $$\sum_{i=1}^{\sigma }x_{i}=x_{1}+x_{2}+x_{3}+\ldots +x_{q}=1\;x_{i}\geq 0$$

where, the “σ” parameter refers to the number of components varying in the mixture and $x_{i}$ is the proportion of each component. In this study, for each mixture design σ = 3, $x_{1}=Ce$, $x_{2}=Pd$ and $x_{3}=Ni$. Consequently, the limits of each compound are:

(3) 0.98≤Ce≤1.00

(4) 0≤Pd≤0.02

(5) 0≤Ni≤0.02

Specifically, for a cubic model, it is important to evaluate the maximum and minimum concentration for each compound, as well as other points at which the three components of the mixture are related according to the rule of experiments. Using a special cubic regression, the model for the asphaltene conversion was established. The regression model equations are as follows:

(6) $$\alpha_{m}=\beta_{1}x_{1}^{\prime }+\beta_{2}x_{2}^{\prime }+\beta_{3}x_{3}^{\prime }+\beta_{12}x_{1}^{\prime }x_{2}^{\prime }+\beta_{13}x_{1}^{\prime }x_{3}^{\prime }+\beta_{123}x_{1}^{\prime }x_{2}^{\prime }x_{3}^{\prime }$$

(7) $$x_{i}^{\prime }=\frac{x_{i}-L_{i}}{1-L}$$

where $\alpha_{m}$ is the conversion of asphaltenes during the steam gasification, $\beta_{i}$, $\beta_{ij}$ and $\beta_{ijk}$ are the coefficients of the linear terms, binary mixtures of nonadditive components, and ternary mixture of nonadditive components, respectively. In the Expression (7), $L_{i}$ is the lower limit of each component, L is the sum of the lower boundaries and $x_{i}^{\prime }$ is a pseudo-component of $x_{i}$ and is used because of the restrictions mentioned in Equations (3)–(5).

### 3.2. Solid-Liquid Equilibrium (SLE) Model

From the theory of association suggested by Talu and Maunier [54] for the self-association and molecular adsorption on micropores, the solid-liquid equilibrium model (SLE) is used to evaluate the adsorption of asphaltenes onto nanoparticles [55]. The expression given by the model is as follows:

(8) $$C=\frac{\psi H}{1+K\psi }e^{\left(\frac{\psi }{q_{m}A}\right)}$$

where K (g·g^−1^) indicates the degree of asphaltenes molecules self-association over the active sites of the nanoparticles surface, H (mg·g^−1^) is an indicator of the adsorption affinity of asphaltenes onto a solid surface, A the surface area (m^2^·g^−1^), $q_{m}$ (mg·m^−2^) is the maximum adsorption capacity of asphaltenes, and ψ defined by:

(9) $$\psi =\frac{-1+\sqrt{1+4K\xi }}{2K}$$

where ξ is given by:

(10) $$\xi =\frac{q_{m}q}{\left(q_{m}-q\right)}A$$

### 3.3. Estimation of Activation Energy and Reaction Kinetic Rate

The effective activation energy ($E_{\alpha }$) can be calculated following the isothermal procedure [28] according to the following Equation (11):

(11) $$\frac{d\alpha }{dt}=K_{\alpha }\exp\left(-\frac{E_{\alpha }}{RT}\right)f\left(\alpha \right)$$

where, α is the conversion degree and is equal to $\left(m_{o}-m_{t})/(m_{o}-m_{f}\right)$; $m_{o}$, $m_{t}$ and $m_{f}$ are the initial mass of the sample, the current mass of the sample at a determined time t, and final mass of the sample, respectively. $K_{\alpha }$ (s^−1^) is the pre-exponential factor, $E_{\alpha }$ (kJ·mol^−1^) is the effective activation energy for a constant degree of conversion, R (J·mol^−1^·K^−1^) is the ideal gas constant, T (K) is the reaction temperature, f(α) is the reaction mechanism function, and dα/dt is the reaction rate. As the analysis was performed under isothermal conditions, the separation of variables and the integration of the Equation (11) is as follows:

(12) g(α)=∫0αdαf(α)=∫0tKαexp(−EαRT)dt=Kαexp(−EαRT)t

Assuming the value of $E_{\alpha }$ constant and taking the natural logarithm for both sides, the Equation (12) is written as (13):

(13) $$ln\left(t_{a,i}\right)=ln\left(\frac{g\left(\alpha \right)}{K_{\alpha }}\right)+\frac{E_{\alpha }}{RTi}$$

The $E_{\alpha }$ is calculated from the slope of the Equation (13) obtained through the plot $\ln(t_{a,i})$ vs. $1/T_{i}$. The subscript i is introduced to indicate different isothermal temperature conditions and $t_{a,i}$ is the time for the reaction to reach a conversion (α) at a given temperature. On the other hand, the slope of Equation (13) gives an approximation of the reaction kinetics and has the next form:

(14) $$ln\left(\frac{g\left(\alpha \right)}{K_{\alpha }}\right)=ln\left(\frac{\int_{0}^{\alpha }\frac{d\alpha }{f(\alpha )}}{K_{\alpha }}\right)=\ln\left(\int_{0}^{\alpha }\frac{d\alpha }{K_{\alpha }(\alpha )}\right)$$

mathematically,

(15) $$-ln\left(\frac{\int_{0}^{\alpha }\frac{d\alpha }{f(\alpha )}}{K_{\alpha }}\right)=ln\left(\frac{1}{\int_{0}^{\alpha }\frac{d\alpha }{K_{\alpha }(\alpha )}}\right)$$

The above equation is solved from the harmonic mean of the reaction rate. This equation is applied as follows:

(16) $$H_{x}=\frac{n}{\sum_{i=1}^{n}\frac{1}{{\left(x\right)}_{i}}}$$

where, $H_{x}$ is the reaction rate, and x=dα/dt, being dα/dt>0. The Equation (13) can be described as the Equation (17) applying the following consideration $\sum_{i=1}^{n}\frac{1}{{\left(\frac{d\alpha }{dt}\right)}_{i}}=k^{\prime }\int_{0}^{\alpha }\frac{1}{\frac{d\alpha }{dt}}d\alpha$

(17) $$H_{\frac{d\alpha }{dt}}=\frac{Kexp\left(-\frac{E_{\alpha }}{RT}\right)}{\frac{g\left(\alpha \right)}{K_{\alpha }}}$$

where $K=n/k^{\prime }$. Taking the natural logarithm of both sides

(18) $$\left(\frac{H_{\frac{d\alpha }{dt}}}{K}\right)=-\ln\left(\frac{g\left(\alpha \right)}{K_{\alpha }}\right)-\frac{E}{RT}$$

Then, comparing the Equations (13) and (18).

(19) $$\ln\left(t_{a,i}\right)=\ln\left(\frac{H_{\frac{d\alpha }{dt}}}{K}\right)$$

(20) $$H_{\frac{d\alpha }{dt}}=\frac{K^{*}}{t_{a,i}}$$

From the estimation of the harmonic mean, the reciprocal of the reaction time and a $K^{*}$ parameter that may depend on the conversion is obtained.

## 4. Results and Discussion

### 4.1. Nanoparticle Characterization

The surface area of the ceria nanoparticles was estimated in 65 m^2^·g^−1^ ± 2 m^2^·g^−1^. Once the metal oxides was added to the nanoparticle, the surface area decreased slightly as the percentage of the TEO increased. However, the surface area is not affected considerably as the support material is non-porous, and there are no pores that can be blocked.

**Figure 1:**
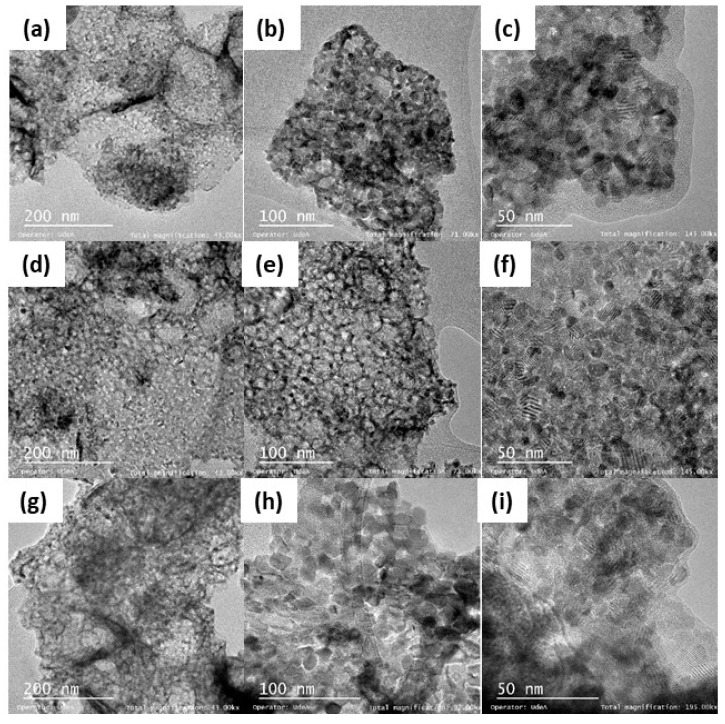
HR-TEM micrographs of (**a**–**c**) CeNi1Pd1, (**d**–**f**) CeCo1Pd1 and (**g**–**i**) CeFe1Pd1 nanoparticles at different magnifications.

The DLS results showed that the mean hydrodynamic diameter of the CeO_2_ nanoparticles was 22 nm, confirming its nanometric nature in agreement with the value reported by the provider. Figure 1 shows the TEM micrographs of CeNi1Pd1, CeCo1Pd1 and CeFe1Pd1 nanoparticles, where an undefined morphology of the support material and crystallization planes in the nanocrystals of the metal oxides are observed. The sizes of the metal oxide crystals and the dispersion thereof on the surface of the support are shown in Table 2. The dispersion of the Pd crystals increased in the order Fe < Co < Ni, while the dispersion of the different metals followed the trend Fe < Ni < Co. In general, for all samples, a smaller crystal size implies a greater dispersion of the same on the surface of the nanoparticle. These results show that the employed metals have a synergistic effect, which avoided sintering processes and therefore generated a less heterogeneous surface due to the high dispersion of these crystals in the support. This could be due to the migration and coalescence of the crystals are significant when the material is heated above the Tamman’s temperature, where the oxides manage to form thermally induced vacancies, called Schottky and Frenkel defects [56,57]. In this sense, the Tamman’s temperatures of Ni [58], Fe [59], and Co [60] are approximately 690 °C, 800 °C and 880 °C, respectively, indicating that the movement of the Co atoms by diffusion over the support surface required higher temperature and energy regarding Ni and Fe. Hence, it can be inferred that higher mobility of the Ni over the support surface would inhibit the Pd mobility, resulting in an alleviated sintering characteristic of complex materials with noble metals [13].

### 4.2. Asphaltene Adsorption onto Nanoparticles

**Figure 2:**
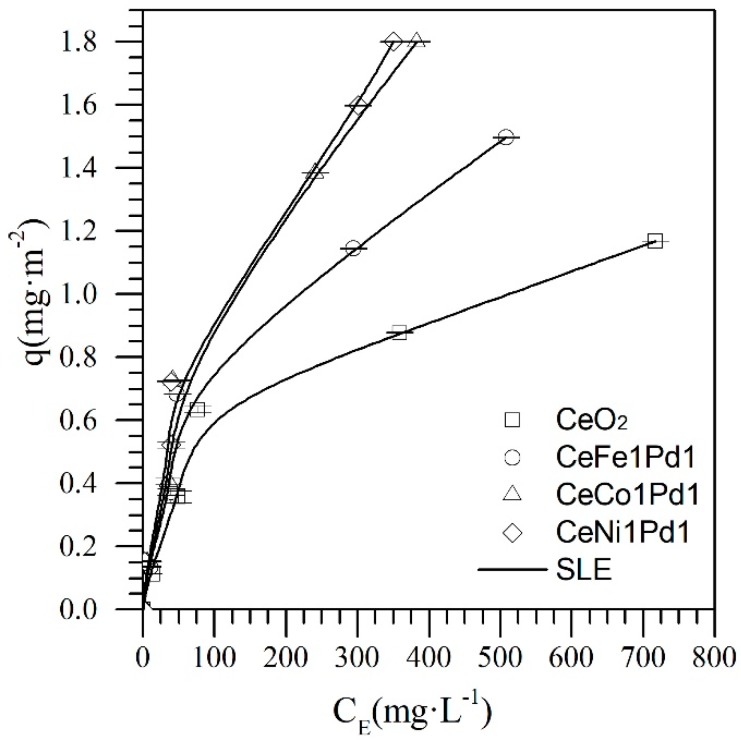
Adsorption isotherms of *n*-C_7_ asphaltenes onto CeO_2_ nanoparticles and CeO_2_ nanoparticles functionalized with Ni, Fe, Co and Pd at 25 °C. Adsorption isotherms were obtained for *n*-C_7_ asphaltene concentrations from 100 mg·L^−1^ to 1500 mg·L^−1^ in toluene for a fixed dosage of nanoparticles of 100 mg per 10 mL of solution. The symbols are experimental data, and the solid lines are from the SLE model.

First, the adsorption isotherms of *n*-C_7_ asphaltenes were obtained for the CeO_2_ support, and the CeO_2_ nanoparticles functionalized with different TEO at a fixed concentration of mass fraction of 1% and 1% of PdO over the ceria surface (CeNi1Pd1, CeCo1Pd1 and CeFe1Pd1). From these nanoparticles, the best TEO-Pd functionalizing couple is selected for performing the SCMD of experiments. Figure 2 shows the adsorption isotherms at 25 °C for the CeNi1Pd, CeCo1Pd1, and CeFe1Pd1 nanoparticles together with the fit of the SLE model.

According to the International Union of Pure and Applied Chemistry (IUPAC), the obtained adsorption isotherms are type Ib [61], which is in agreement with previous studies [13,15,22,29,30,31,37,62]. In all cases, the asphaltene adsorption was higher for the bi-metallic nanoparticles than for the cerium oxide support. In addition, between the three bimetallic systems, the adsorption increased in the order CeO_2_ < CeFe1Pd1 < CeCo1Pd1 < CeNi1Pd1. This trend could be due to the high dispersion of metals on the surface of the CeNi1Pd1 sample regarding the other adsorbents that lead to stronger intermolecular forces that are generated between heteroatoms (HA) and functional groups of the asphaltenes.

Additionally, these results are corroborated with the parameters obtained by the SLE model, where the value of H decreased in the order CeO_2_ > CeFe1Pd1 > CeCo1Pd1 > CeNi1Pd1, indicating that there was a higher affinity between the asphaltenes and the bimetallic nanoparticles. This TEO-based catalyst showed a good capability to interact with carbonaceous molecules and activate the C-C, C–O, C–N, and C–H bonds that allow to use them in reactions such as cracking, isomerization, and hydroprocessing, among others. According to the adsorption experiments and the values of H from SLE model, the affinity for asphaltene uptake for the different nanoparticles changes due to the presence of the different TEO used, showing a greater affinity for the Ni metal oxides than for the other systems. Hence, it can be established that these TEO/CeO_2_ hybrid materials interact with the asphaltene molecules via coordinated bonds (HA-TE) [63].

It is well known that the organic functional groups containing HA (O, N, and S) are Lewis bases due to the electron lone pairs located on them. Therefore, this family of molecules can form coordinate bonds by interacting with Lewis acids, such as transition elements (TE) with adequate orbitals and strength. It is expected that the Lewis acidity changes as the periodic group increases for the different elements. This can be explained by the increase of the effective nuclear charge (Fe < Co < Ni) [64]. On the other hand, the interaction between TE and Pd shows synergetic effects that improve the catalytic performances.

**Figure 3:**
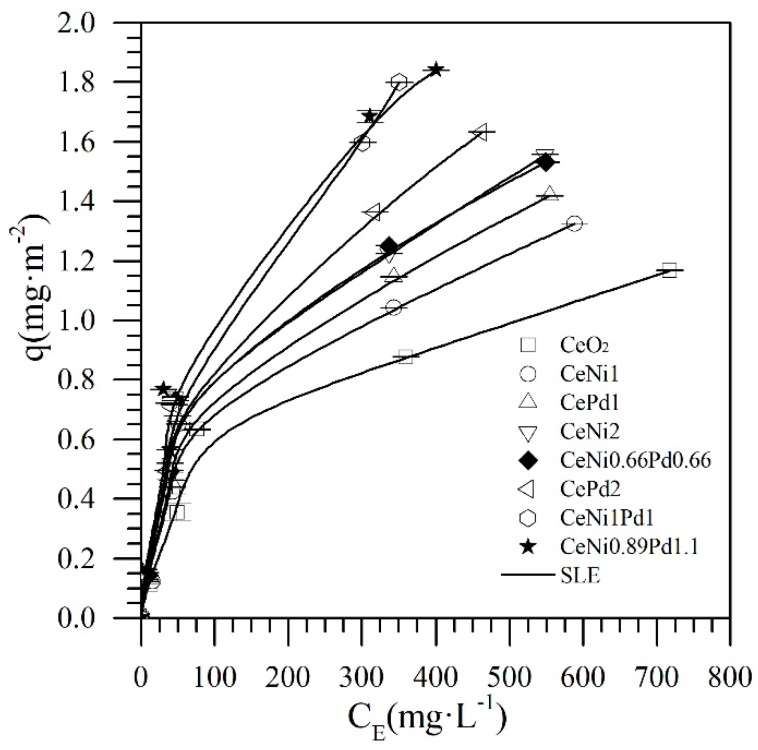
Adsorption isotherms at 25 °C of *n*-C_7_ asphaltenes onto CeO_2_ nanoparticles and CeO_2_ nanoparticles functionalized with different mass fractions of Ni and/or Pd up to 2%. Adsorption isotherms were obtained for *n*-C_7_ asphaltene concentrations from 100 mg·L^−1^ to 1500 mg·L^−1^ in toluene for a fixed dosage of nanoparticles of 100 mg per 10 mL of solution. The symbols are experimental data, and the solid lines are from the SLE model.

Accordingly, the Ni–Pd couple was selected to optimize the metal concentrations over the CeO_2_ support according to the simplex-centroid mixture design of experiments. Figure 3 shows the adsorption isotherms at 25 °C of *n*-C_7_ asphaltenes onto the nanoparticles established in the SCMD. In general, all samples showed high adsorption affinity for *n*-C_7_ asphaltenes. The asphaltene uptake follows the order CeNi1Pd1 > CePd2 > CeNi0.66Pd0.66 > CeNi2 > CePd1 > CeNi1 > CeO_2_. Table 3 summarizes the estimated SLE parameters for all nanoparticles evaluated. The values for the *H* parameter (related to the affinity for the adsorption) increase in the order CeNi1Pd1 < CePd2 < CeNi0.66Pd0.66 < CeNi2 < CePd1 < CeNi1 < CeO_2_, corroborating that the loading of the metal on the support surface matters insofar as H is smaller for the CePd2 and CeNi2 nanoparticles than for CePd1 and CeNi1 materials.

Also, the value of H is lower for the bimetallic system (CeNi1Pd1), indicating that the properties that the material acquires by the two metal oxides increase its adsorptive capacity and affinity for *n*-C_7_ asphaltenes. On the other hand, the K parameter follows the same trend as H, indicating the highest association rate of asphaltenes molecules once the primary sites were occupied for nanoparticle support without metals onto its surface. This suggests that ceria supported TEO nanoparticles are more prone to inhibit the asphaltenes self-association over its surface than the support [14,31,62].

### 4.3. Catalytic Steam Gasification of n-C_7_ Asphaltenes

#### 4.3.1. Mass Loss Analysis

**Figure 4:**
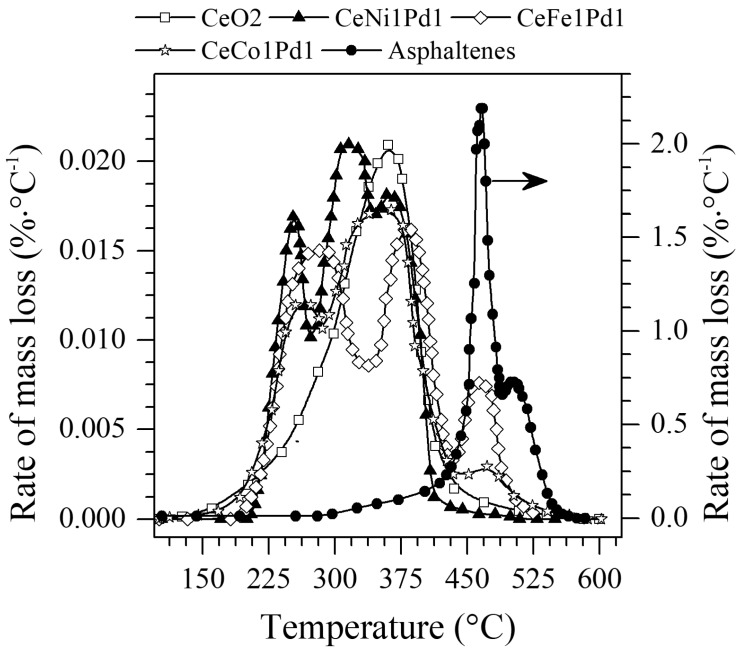
The rate of mass loss as a function of the temperature for steam gasification of *n*-C_7_ asphaltenes in the absence and presence of CeO_2_, CeNi1Pd1, CeCo1Pd1, and CeFe1Pd1 nanoparticles. Nitrogen flow rate = 100 mL·min^−1^, H_2_O_(g)_ flow rate = 6.30 mL·min^−1^, heating rate = 20 °C·min^−1^ and *n*-C_7_ asphaltene loading 0.2 mg·m^−2^.

The selected nanoparticles were tested in an atmosphere of N_2_ saturated with H_2_O_(g)_ for asphaltenes catalytic steam gasification. Figure 4 shows the rate of mass loss of *n*-C_7_ asphaltenes in the presence and absence of CeO_2_ nanoparticles, and CeO_2_ nanoparticles functionalized with Ni, Fe, Co, and Pd metal oxides. Figure 4 reveals that for virgin *n*-C_7_ asphaltenes, the decomposition occurs between 400 °C and 550 °C. When asphaltenes are adsorbed onto nanoparticles, gasification of *n*-C_7_ asphaltenes appears to happen at much lower temperatures.

The temperature range evaluated will be divided into three regions, the first being the low-temperature region (LTR) whose temperature values vary between 200–250 °C. The second region of medium temperature (MTR) will be from 251 °C to 450 °C, and from 451 °C to 600 °C will be the high-temperature region (HTR). From the rate of mass loss plot, it can be observed that CeO_2_ nanoparticles without functionalization reduce the decomposition temperature of *n*-C_7_ asphaltenes from approximately 455 °C to 370 °C. However, all the functionalized samples manage to reduce the *n*-C_7_ asphaltenes decomposition temperature from HTR to LTR in the order Ni > Co > Fe. However, this decomposition is generated in several stages for the three cases, indicating a distribution of asphaltene sizes (low, medium and high molecular weight). The components with lower molecular weight decompose at lower temperatures, and these values vary for all nanoparticles due to the transferring electrons that different TEO have thanks to their charges on the surface of CeO_2_.

The decomposition of *n*-C_7_ asphaltenes over CeNi1Pd1 nanoparticles begins at 220 °C in the LTR region, continues at 310 °C in the MTR region where it decomposes larger chains of hydrocarbons and ends in this same region at 375 °C. This means that during the gasification process the heavier compounds were cracked in lower molecular weight hydrocarbon chains either by the loss of heteroatoms in the breakage of bonds (C–S, C–N, C–O) or the breakdown of C–C and C–H bonds. For the CeCo1Pd1 and CeFe1Pd1 nanoparticles, they produce decomposition of *n*-C_7_ asphaltenes in the three regions, at 230 °C, 350 °C, 455 °C and 250 °C, 370 °C, and 455 °C, respectively. Between these two systems, the CeCo1Pd1 system in the HTR region produces a lower mass loss compared to CeFe1Pd1. It is clear that among the three systems evaluated, the system formed by Ni and Pd had a synergistic effect reflected in its better performance for the decomposition of *n*-C_7_ asphaltenes in the LTR region.

Additionally, differences in the intensity of the peaks in the different regions could be due to the suppression of addition reactions of the *n*-C_7_ asphaltenes after decomposition. Samples that show peaks with lower intensities in the MTR and HTR have a higher catalytic activity, as they would promote an earlier decomposition independently of the molecular weight of the asphaltene adsorbed.

**Figure 5:**
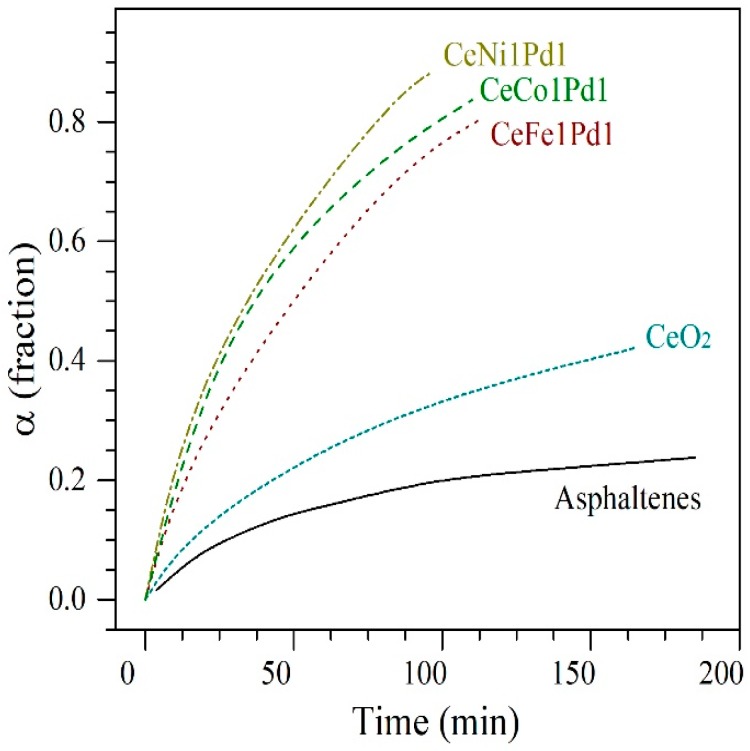
Isothermal conversion (α) for steam gasification of *n*-C_7_ asphaltenes in the absence (360 °C) and presence (240 °C) of CeO_2_, CeNi1Pd1, CeCo1Pd1, and CeFe1Pd1 nanoparticles. Nitrogen flow rate = 100 mL·min^−1^, H_2_O_(g)_ flow rate = 6.30 mL·min^−1^ and *n*-C_7_ asphaltene loading 0.2 mg·m^−2^.

Figure 5 shows the conversion or the extent of reaction (α) of *n*-C_7_ asphaltenes in the presence and absence of the employed nanoparticles as a function of time at isothermal conditions of 240 °C. As it can be seen in Figure 5, the presence of nanoparticles generates a catalytic effect reflected in the decomposition time or reaction velocity, which is lowest for CeNi1Pd1 nanoparticles, followed by CeCo1Pd1, CeFe1Pd1, and CeO_2_. However, an important change was achieved in the conversion concerning virgin asphaltenes for all samples.

The best catalytic is obtained for the CeNi1Pd1 nanoparticles with an 89% of asphaltene conversion in less than 100 min at 240 °C. It is important to note that the differences in the catalytic activity are related to the synergistic effect of the TE on the surface of the CeO_2_ nanoparticles, and the interactions between asphaltenes-nanoparticles, because the asphaltenes, being non-pure compounds, could have different selectivity for different compounds. The higher catalytic activity of the Ni-Pd couple could be due to the inhibition of the growth of the PdO nanocrystal and the enhanced dispersion over the CeO_2_ surface.

#### 4.3.2. Analysis of the Gaseous Products Evolved during the Steam Gasification Process

**Figure 6:**
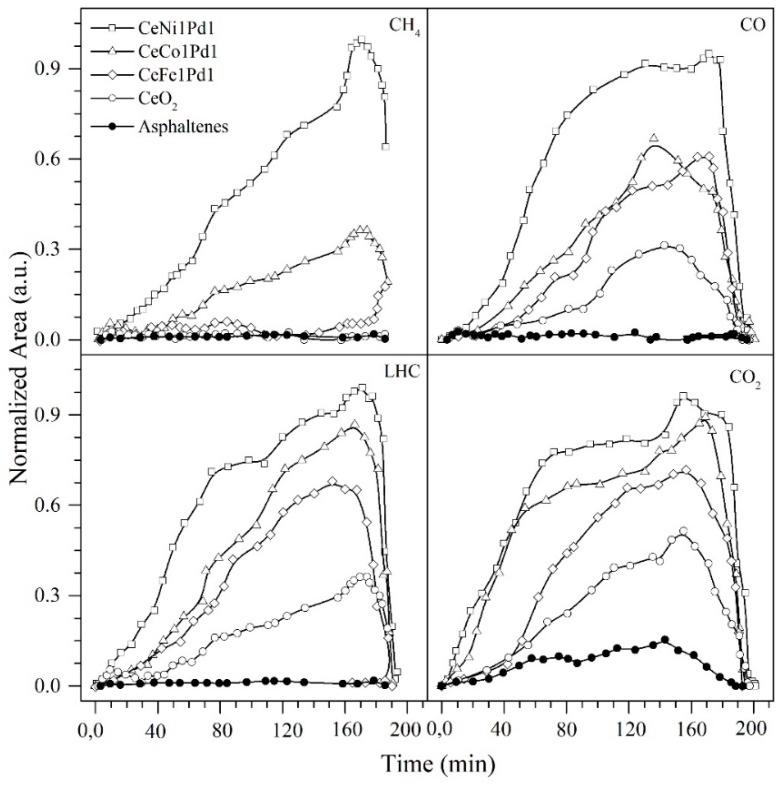
Evolution profiles of gaseous products during steam gasification under isothermal conditions at 240 °C of *n*-C_7_ asphaltenes in the presence and absence of the selected catalysts. Nitrogen flow rate = 100 mL·min^−1^, H_2_O_(g)_ flow rate = 6.30 mL·min^−1^ and *n*-C_7_ asphaltene loading 0.2 mg·m^−2^.

The evolution of gaseous products during the gasification process of virgin asphaltenes and asphaltenes adsorbed onto CeO_2_, CeNi1Pd1, CeCo1Pd1, and CeFe1Pd1 nanoparticles is shown in Figure 6. The gaseous products of asphaltenes gasification were evaluated using an FTIR device coupled to the TGA under isothermal conditions of 240 °C. During the steam gasification of *n*-C_7_ asphaltenes, a series of reactions occur such as water-gas-shift (WGS) reaction, partial oxidation reaction, Boudouard reaction, methanation reaction, and steam reforming reaction, among others, generating as main products CO, CO_2_, CH_4_, NO_X_, and light hydrocarbons (LHC). Results are normalized based on the signal with the highest intensity that corresponds to the gases production for CeNi1Pd1, in all cases. The gases that are being produced are initially due to the catalytic effect of CeO_2_ and its redox cycle (${\mathrm{Ce}}^{4+}/{\mathrm{Ce}}^{3+}$) that allows the oxygen adsorption under oxidation conditions and its release under reduced conditions, as is shown in Equations (21) and (22):

(21) CeO2−δ+12δ O2⇋CeO2

(22) CeO2⇒CeCex+2OOx↔CeCe•+12VO″+12O2

Bimetallic systems increased gas production in the following order (Fe < Co < Ni). The high performance of the CeNi1Pd1 system is mainly due to the influence of nickel oxides as a dispersant material together with cerium as an active support material in the acceleration of the production of the WGS reaction at low temperatures (240 °C) due to the oxygen anion vacancy on the surface [47]. This reaction is generally associated to two mechanisms in which the cerium oxide generates a catalytic effect; namely, (a) associative mechanism mediated by methanoate species and (b) redox mechanism, which is widely favored by the addition of noble metals in the support. Hydroxyl groups (OH) formed by the reaction between H_2_O and partially reduced ceria oxygen vacancies react with CO to form methanoate species of bridge. Above 170 °C, these species are transformed into bidentate formates to decompose into final products of CO_2_ and H_2_ [65]. Vignatti et al. [66] proposed a mechanism mediated by methanoate species to explain the catalytic behavior of the ceria with the addition of noble transition elements (TE) like Pd, according to Equations (23)–(26), where θ refers to the support and σ represents the adsorption sites in the TE [44,45,46]:

(23) $${\mathrm{CO}}_{\left(g\right)}+\sigma \to {\mathrm{CO}}_{\mathrm{ads}}$$

(24) $${\mathrm{CO}}_{\mathrm{ads}}+\theta -\mathrm{OH}\to \theta -\mathrm{HCOO}+\sigma$$

(25) $$\theta +H_{2}O_{\left(g\right)}\to \theta -H_{2}O$$

(26) $$\theta -\mathrm{HCOO}+\theta {-H}_{2}O\to {\mathrm{CO}}_{2\left(g\right)}+H_{2\left(g\right)}+\theta +\theta -\mathrm{OH}$$

According to the redox reaction mechanism, the CO is adsorbed on the active sites of the TE to react with the cerium oxide, reducing it to cerium (III) oxide (schematized as CeO_2-*δ*_, see Equations (21) and (22)). This reduction generates oxygen vacancies or defects, that on contact with water molecules, produces OH groups and eventually H_2_ (Equations (23)–(26)). Further, it is important to emphasize the role of TE in activating CO, and forming oxygen vacancies in the TE/ceria interface [39,40], as follows:

(27) $$\mathrm{CO}+\sigma \to {\mathrm{CO}}_{\mathrm{ads}}$$

(28) $${\mathrm{CO}}_{\mathrm{ads}}+2{\mathrm{CeO}}_{2}\to {\mathrm{CO}}_{2}+{\mathrm{Ce}}_{2}O_{3}+\sigma$$

(29) $${\mathrm{Ce}}_{2}O_{3}{+H}_{2}O\to 2{\mathrm{CeO}}_{2}{+H}_{2}$$

Hence, the CO is produced through the reaction of free radicals with the oxygen released from O-containing functional groups in the asphaltene structures. Further, the production of this gas generates the production of H_2_ due to the catalytic activity of CeO_2_ loaded with TEO on the water-gas shift reaction and CO oxidation. The CeO_2_ role is not limited to storage of oxygen since it allows the transfer of oxygen from ceria to the TEO interface and re-oxidation of ceria through the adsorption of CO on the active sites of the TEO, as shown in Equations (27)–(29). On the other hand, there are two main oxygen sources to which the CO_2_ production can be attributed: (i) the oxygen content of the asphaltenes and (ii) lattice oxygen from the nanoparticles. Gas production increases in the order virgin asphaltenes < CeO_2_ < CeFe1Pd1 < CeCo1Pd1 < CeNi1Pd1. For all cases, the evolution profile constantly increases with time. The decrease in time at which the gaseous production begins with the addition of the CeO_2_, CeNi1Pd1, CeCo1Pd1, and CeFe1Pd1 nanoparticles, corroborates the catalytic activity of the same, showing an improvement in the CeNi1Pd1 nanoparticle over the others.

#### 4.3.3. Coke Yield

**Figure 7:**
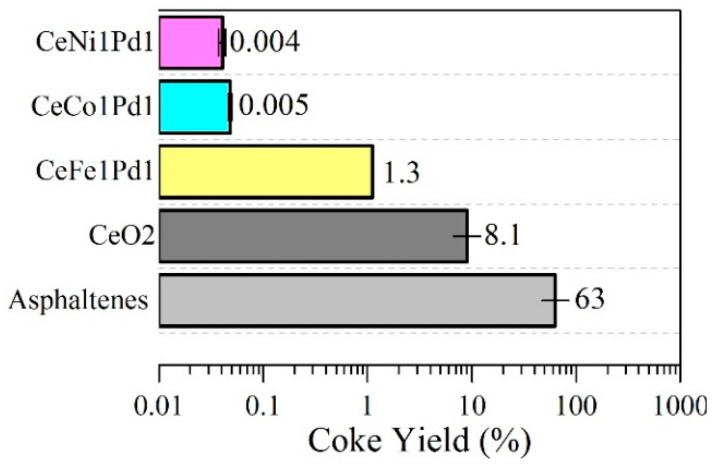
Coke yield for *n*-C_7_ asphaltenes decomposition under steam gasification in the absence and presence of CeO_2_, CeNi1Pd1, CeCo1Pd1, and CeFe1Pd1 nanoparticles. Nitrogen flow rate = 100 mL∙min^−1^, H_2_O_(g)_ flow rate = 6.30 mL∙min^−1^, heating rate = 20 °C∙min^−1^ and asphaltene loading 0.2 mg∙m^−2^.

For the asphaltenes in the presence and absence of CeO_2_, CeNi1Pd, CeCo1Pd1, and CeFe1Pd1, the coke yield was evaluated under an air atmosphere from 100 °C to 600 °C for 20 °C·min^−1^, with a flow rate of 100 mL·min^−1^. Figure 7 shows the results obtained for the coke yield of the samples evaluated. For virgin *n*-C_7_ asphaltenes, the coke yield was estimated in a mass fraction of 63% ± 2% and for *n*-C_7_ asphaltenes adsorbed onto the CeO_2_ nanoparticles surface, the coke yield was 8.1%. In the case of the functionalized nanoparticles, the coke yield follows the trend CeFe1Pd1 > CeCo1Pd1 ≅ CeNi1Pd1 with an inhibition of the coke production of more than 99%, regarding the CeO_2_ support.

In general, and through the analysis performed in each of the tests, the nanoparticle that has a better performance regarding catalytic activity refers to CeNi1Pd1. Further, the Ni-Pd couple is selected for the following experiments. It is important to emphasize at this point that the CeO_2_, CeFe1Pd1, and CeCo1Pd1 nanoparticles also have a good catalytic effect in the reactions that generate the asphaltenes decomposition.

### 4.4. Effect of n-C_7_ Asphaltene Amount Adsorbed on the Decomposition Temperature

**Figure 8:**
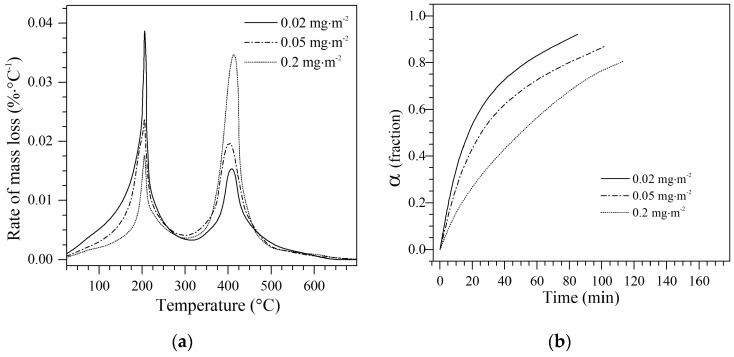
(**a**) Plot of rate of mass loss and (**b**) isothermal conversion (α) at 220 °C for asphaltene loadings of 0.2 mg·m^−2^, 0.05 mg·m^−2^, and 0.02 mg·m^−2^ over CeNi1Pd1 nanoparticles. Nitrogen flow rate = 100 mL·min^−1^, H_2_O_(g)_ flow rate = 6.30 mL·min^−1^. The samples were heated at 20 °C∙min^−1^ until 220 °C (according to the first decomposition peak) where isothermal conditions were fixed until no significant changes in the mass loss were observed, and finally heated again up to 700 °C at the same heating rate.

According to the results, the CeNi1Pd1 sample was used to evaluate the effect of the amount adsorbed of *n*-C_7_ asphaltenes on the decomposition temperature. For this analysis, three different loads of asphaltenes of 0.2 mg·m^−2^, 0.05 mg·m^−2^, and 0.02 mg·m^−2^ were evaluated. In this case, the samples were heated at 20 °C∙min^−1^ until 220 °C (according to the first decomposition peak for this sample in Figure 4) where isothermal conditions were fixed until no significant changes in the mass loss were observed, and finally heated again up to 700 °C at the same heating rate. Figure 8 shows (a) the rate of mass loss and (b) isothermal conversion at 220 °C for the CeNi1Pd1 nanoparticles with different asphaltene amounts adsorbed. The three samples exhibit two peaks of mass loss. However, as the loading of asphaltenes decreases, the intensity of the first peak increases and the second decreases. This effect is possibly due to the asphaltenes are adsorbed first on the TEO active sites and not over the ceria active sites. Also, a lower amount of asphaltenes adsorbed can lead to lower self-association over the active sites, leading to a lower aggregate size over the catalyst surface and a less amount of energy needed for decomposition. As can be observed from Figure 8b, when the amount of asphaltenes decreases, the conversion increases, since a greater number of active sites free for the reaction are available.

### 4.5. Maximization of Conversion of Asphaltenes during Steam Gasification through an SCMD

#### 4.5.1. Isothermal Conversion of Asphaltenes Adsorbed on Nanoparticles from SCMD

The maximum conversion of the asphaltenes adsorbed on nanoparticles was used as a response variable ($\alpha_{m}$) when performing the maximization. The process was carried out for the best system among the three functionalizing couples evaluated, (i.e., CeNi1Pd1) for an amount adsorbed of asphaltenes of 0.02 g·m^−2^ under isothermal conditions at 220 °C according to the results in Figure 8. The STATGRAPHICS Centurion XVI software was used to obtain the statistical results, and the process was validated using the special cubic model with a $R^{2}$ > 0.99. In this way, it is possible to predict the conversion of asphaltenes for any concentration of Ce, Pd, and Ni in the evaluated range. Table S1 of the Supplementary Materials document lists the values of β for the model of the conversion as a function of the pseudo-components Ce, Ni, and Pd. The optimal point for conversion maximization is found for dosages of mass fractions of 1.1% and 0.89% of palladium and nickel, respectively (CeNi0.89Pd1.1). For this sample, an adsorption isotherm was constructed as previously showed in Figure 3. As can be seen, the CeNi0.89Pd1.1 nanoparticle has a better performance than all the samples except for CeNi1Pd1 and CePd2 for $C_{0}$ < 500 mg·L^−1^.

**Figure 9:**
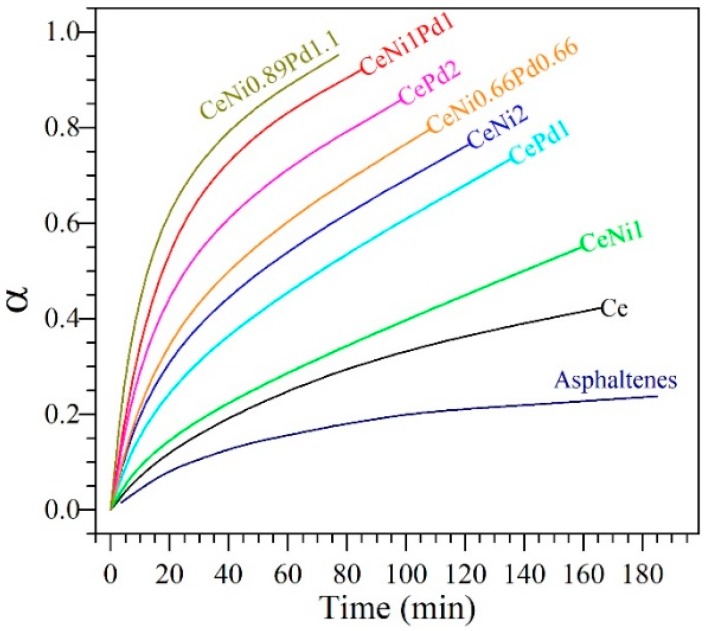
Isothermal conversion (α) for steam gasification of *n*-C_7_ asphaltenes in the absence (360 °C) and presence (220 °C) of CeO_2_, CeNi1, CeNi2, CePd1, CePd2, CeNi0.66Pd0.66, CeNi1Pd1, and CeNi0.89Pd1.1 (optimized sample) nanoparticles. The nanoparticles are selected from a simplex-centroid mixture design of experiments for maximizing the asphaltene conversion. Nitrogen flow rate = 100 mL·min^−1^, H_2_O_(g)_ flow rate = 6.30 mL·min^−1^ and *n*-C_7_ asphaltene loading 0.02 mg·m^−2^.

Figure 9 shows the asphaltene conversion using CeNi0.89Pd1.1 nanoparticles compared to all samples evaluated from the SCMD, where it can be observed a reduction in the time of reaction for asphaltene decomposition for the optimized sample, including the CeNi1Pd1 material. Also, the results in Figure 9 indicate that a better catalytic activity is obtained for bimetallic samples regarding the monometallic nanoparticles. However, the SPd2 shows a better performance than the bimetallic CeNi0.66Pd0.66, indicating that the PdO presence is a key controller parameter in the catalytic activity of the material. These results also corroborate that the crystallite size and dispersion of the active phase lead to a more efficient decomposition. The importance of this optimization lies on the reduction of the TEO percentage to generate a better catalytic effect and a reduction of the Pd concentration in almost 50% regarding the SPd2 sample.

#### 4.5.2. Effective Activation Energy and Kinetics of the Catalytic Steam Gasification of Asphaltenes in the Presence and Absence of Nanoparticles

**Figure 10:**
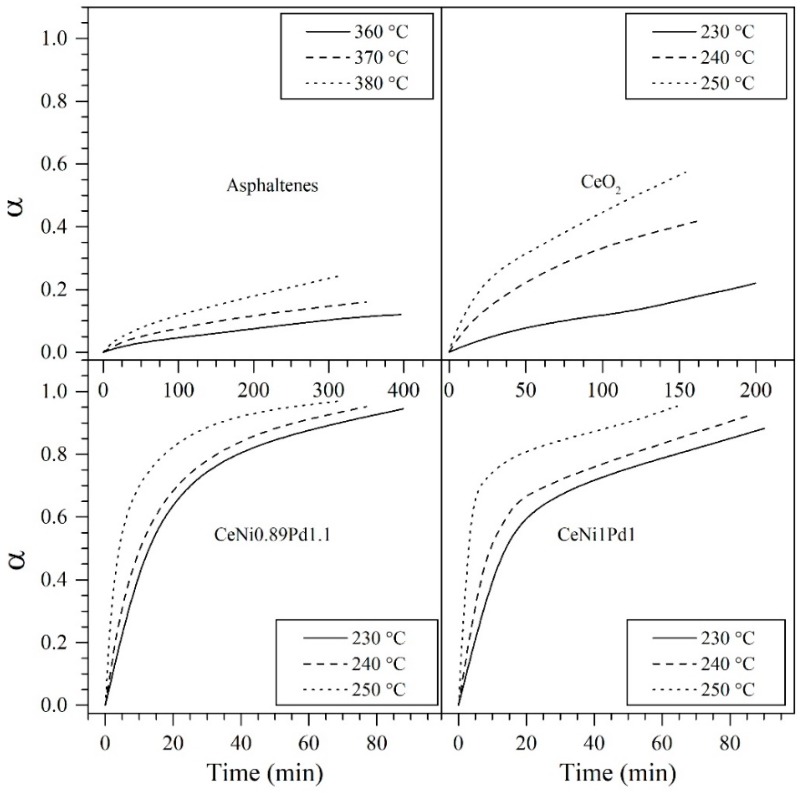
Isothermal conversion (α) at different temperatures for steam gasification of *n*-C_7_ asphaltenes in the absence and presence of CeO_2_, CeNi1Pd1, and CeNi0.89Pd1.1 (optimized sample) nanoparticles. The nanoparticles are selected from a simplex-centroid mixture design of experiments for maximizing the asphaltene conversion. Nitrogen flow rate = 100 mL·min^−1^, H_2_O_(g)_ flow rate = 6.30 mL·min^−1^ and *n*-C_7_ asphaltene loading 0.02 mg·m^−2^.

Thermogravimetric analyses were carried out for asphaltenes adsorbed on all nanoparticles selected from the SCMD at three different temperatures of 230 °C, 240 °C, and 250 °C to obtain information about the catalytic effect of the selected TEO nanoparticles under isothermal conditions regarding effective activation energy and reaction kinetics. In the case of asphaltenes in the absence of nanoparticles, the employed temperatures were 350 °C, 360 °C, and 370 °C. Figure 10 shows the time of isothermal conversion (α) of virgin asphaltenes and adsorbed asphaltenes onto CeO_2_, CeNi1Pd1, and CeNi0.89Pd1.1 at the corresponding temperatures.

**Figure 11:**
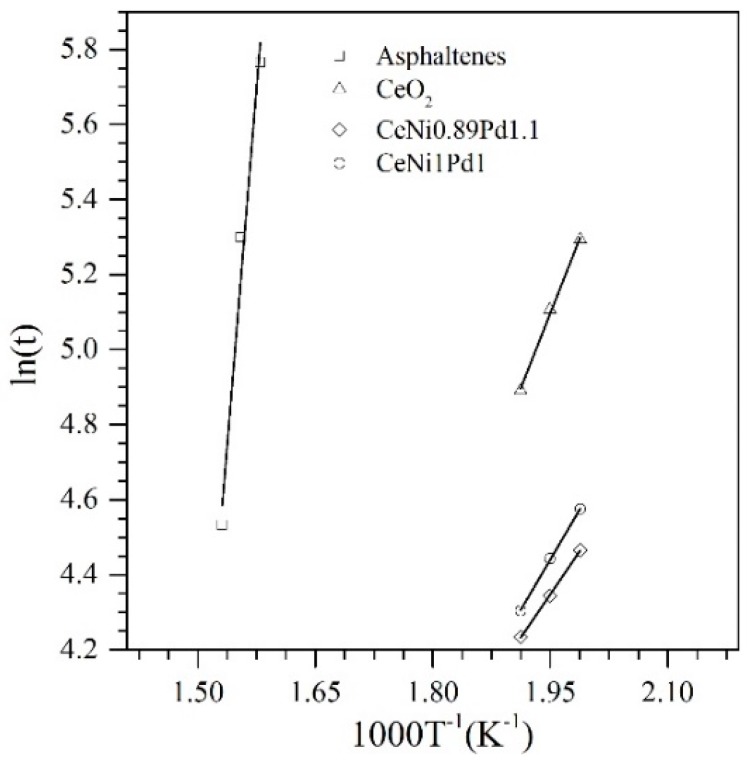
Arrhenius plot for the isothermal model of catalytic and thermal gasification of asphaltenes in the presence and absence of CeO_2_, CeNi1Pd1, and CeNi0.89Pd1.1 (optimized sample) nanoparticles.

As expected, the conversion of asphaltenes increased with the increase in temperature. For the asphaltenes in the presence of CeO_2_ nanoparticles, the asphaltene conversion was lower than 0.6 for the three temperatures evaluated. For the CeNi1Pd1 bimetallic sample, the conversion is 0.9, 0.92 and 0.96 for 230 °C, 240 °C, and 250 °C. In the case of the optimized sample, for all temperatures evaluated, an α = 1 was obtained, indicating that an effective decomposition of asphaltenes can be obtained with this material under typical temperatures of steam injection EOR processes. The effective activation energies ($E_{\alpha }$) were calculated from the slope of the plot $\ln(t_{a,i})$ vs. $1/T_{i}$ in Figure 11, and Table 4 summarizes the results obtained for the $E_{\alpha }$ and reaction kinetics.

For the cases evaluated, it was found that the effective activation energy was lower in all cases in the presence of nanoparticles, indicating a shift in the reaction mechanism. The values of $E_{\alpha }$ followed the trend CeN0.89Pd1.1 < CeNi1Pd < CeO_2_, indicating a higher efficiency in the decomposition of the asphaltenes for the optimized sample, and is corroborated with the values of the kinetic rate that suggest that the reaction occurs faster. Because these values are roughly an estimation of the potential reaction rate achievable without the reaction barrier, it represents an effective variable when comparing the catalytic activity of the nanoparticles. The kinetic reaction rate was faster for CeNi0.89Pd1.1 than CeNi1Pd1, and this last was faster than CeO_2_, demonstrating that the gasification reaction of asphaltenes is affected by the specific chemical elements used, and their proportions.

**Figure 12:**
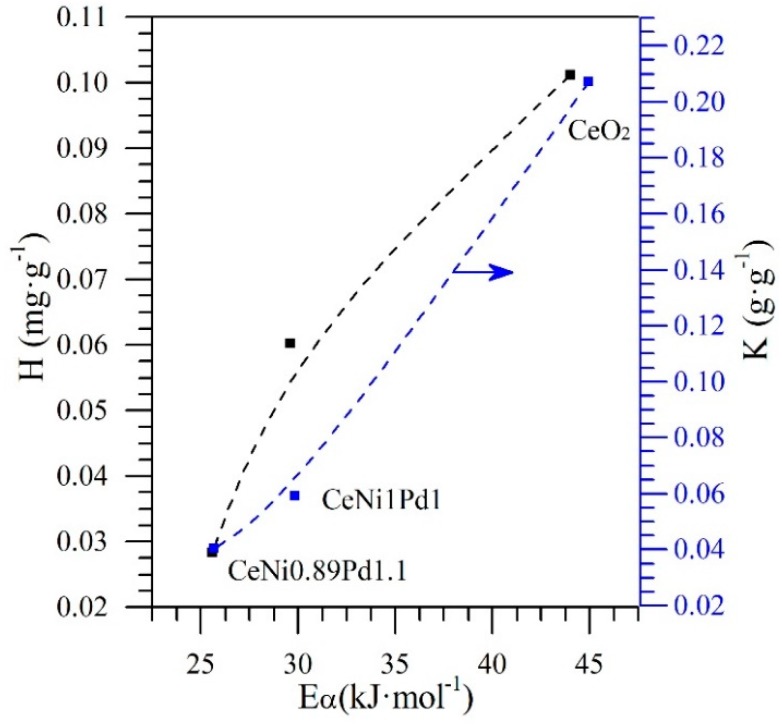
The relationship between Henry’s law constant (*H* parameter of the SLE model), the degree of asphaltene self-association over the active sites of the catalyst (*K* parameter of the SLE model) and the effective activation energies ($E_{\alpha }$) calculated by the isothermal model for the catalytic steam gasification of *n*-C_7_ asphaltenes.

Figure 12 shows the relationship between the adsorptive behavior according to the SLE model parameters and the catalytic activity of the CeO_2_, CeNi1Pd1, and CeNi0.89Pd1.1 systems in terms of the effective activation energy. It can be observed from Figure 12 a direct relationship between Henry’s law constant (adsorption affinity) and the energy required to carry out the catalytic steam gasification of the *n*-C_7_ asphaltenes. The values of $E_{\alpha }$ increased in the order CeO_2_ < CeNi1Pd1 < CeNi0.89Pd1.1, which indicates that there is a synergistic effect between the support and functionalizing agents (NiO and PdO). The presence of these active phases also increases the affinity of the *n*-C_7_ asphaltenes mainly in the active sites belonging to the TEOs. Hence, the values of the H parameter (Table 3) decrease in the same order than $E_{\alpha }$, indicating a direct relationship between the catalytic activity and adsorption capacity of the materials. Similarly, it is observed that the effective activation energy increased as the *K* parameter increased, suggesting that for a higher asphaltene self-association over the active sites of the nanoparticles, higher energy is required for the decomposition. This could be due to the proximity of the asphaltene to the active phase that is lower for systems with multilayers. Also, results indicate that the distribution of the active phase over the CeO_2_ support is a controlling factor for the adsorption and subsequent catalytic decomposition of the asphaltenes. These trends are in agreement with previous studies [13] in which the effective activation energy was related to the adsorption affinity, the degree of asphaltene self-association, and the chemical nature of the support.

## 5. Conclusions

This study looks into the synergistic effect between PdO with NiO, Co_3_O_4_, or Fe_2_O_3_ supported on ceria nanoparticles, to evaluate the catalytic effect on the catalytic steam decomposition of the asphaltenes extracted from an extra heavy oil sample. The evaluated temperatures were lower than 250 °C under isothermal conditions, aiming at reproducing the temperatures of steam injection EOR processes. Regarding the adsorptive capacity of the nanoparticles, this increases with the addition of TEO on its surface. Likewise, these nanoparticles are prone to reduce the degree of self-association of asphaltenes, and increase the affinity of adsorption as confirmed by the values of the parameters K and H of the SLE model. Among the three main systems Ni–Pd, Co–Pd, and Fe–Pd over the ceria support, the CeNi1Pd1 is the one that shows the best results in terms of reducing the temperature of asphaltenes decomposition in steam injection processes and its respective maximum conversion generated under isothermal conditions. Also, the nanoparticles generate an inhibition of the coke formation and, like the other systems evaluated, increase the production of CH_4_, CO, and CO_2_. These results indicate that water gas shift and other reactions are occurring, and hence the production of H_2_ will increase, allowing the hydrogenation of the cracked asphaltene molecules. A simplex-centroid mixture design (SCMD) was performed to optimize the concentration of transition element oxides on the surface of the nanoparticle, obtaining the optimized system CeNi0.89Pd1.1 which effectively generates a conversion of 100% of asphaltenes in less than 90 min at 220 °C, whose value is within the range of temperatures in which a steam injection process is carried out. Results showed that the employed SCMD is able to predict the physicochemical properties in complex systems. The optimized nanoparticles can reduce the effective activation energy in a higher degree than CeNi1Pd1 nanoparticles, as well as showing a faster kinetic rate over the asphaltene decomposition, confirming that different nanoparticles utilized different reaction mechanisms.

Further studies should include the regeneration of the proposed catalysts, the effect of other heavy hydrocarbons such as resins, the effect of high pressure (reservoir conditions), as well as displacement tests for heavy and extra-heavy oil upgrading in porous media. This study should open a broader landscape about the implementation of nanotechnology in the oil and gas industry, and expands the range of application of nanoparticles and nanofluids in thermal enhanced oil recovery operations.

## Figures and Tables

**Table 1:** Nomenclature, mass fraction and the molar fraction of the prepared nanoparticles composed by ceria support (CeO_2_) functionalized with transition metal oxides (Fe_2_O_3_, Co_3_O_4_, NiO and/or PdO).

Sample	Nominal Oxides	Nominal Mass Fraction (%)	Nominal Molar Fraction (%)
CeO_2_	CeO_2_	100.0	100.0
CeNi1Pd1	CeO_2_	98.0	98.8
	NiO	1.0	0.4
	PdO	1.0	0.7
CeFe1Pd1	CeO_2_	98.0	98.4
	Fe_2_O_3_	1.0	0.9
	PdO	1.0	0.7
CeCo1Pd1	CeO_2_	98.0	97.9
	Co_3_O_4_	1.0	1.4
	PdO	1.0	0.7
CeNi2	CeO_2_	98.0	99.1
	NiO	2.0	0.9
CePd2	CeO_2_	98.0	98.6
	PdO	2.0	1.4
CeNi1	CeO_2_	99.0	99.6
	NiO	1.0	0.4
CePd1	CeO_2_	99.0	99.3
	PdO	1.0	0.7
CeNi0.66Pd0.66	CeO_2_	98.7	99.2
	NiO	0.7	0.3
	PdO	0.7	0.5

**Table 2:** Estimated values of surface area (S_BET_), NiO, Fe_2_O_3_, Co_3_O_4_ and PdO mean crystal diameter (dp) and the dispersion over the CeO_2_ support.

Sample	S_BET_ ± 0.1 m^2^·g^−1^	dp (nm ± 0.2 nm)				Dispersion (%)	
		NiO	Co_3_O_4_	Fe_2_O_3_	PdO	Ni/Co/Fe	Pd
CeO_2_	67.0	-	-	-	-	-	-
CeNi1Pd1	63.8	6.4	-	-	3.9	12.7	38.6
CeFe1Pd1	64.1	-	-	5.4	6.9	11.2	12.8
CeCo1Pd1	64.4	-	1.9	-	6.1	18.1	20.4

**Table 3:** Estimated values of the SLE model parameters for the adsorption isotherms of *n*-C_7_ asphaltenes onto CeO_2_ nanoparticles and CeO_2_ nanoparticles functionalized with Ni, Fe, Co, and Pd at 25 °C.

Material	*H* ± 0.02 (mg·g^−1^) × 10^−2^	*K* ± 0.08 (g·g^−1^)	*q_m_* ± 0.01 (g·g^−1^)	*RSM* (%)
CeO_2_	10.12	0.21	0.13	0.02
CePd1	7.19	0.29	0.16	0.03
CeNi1	7.34	0.03	0.15	0.04
CePd2	6.25	0.39	0.18	0.01
CeNi2	6.70	0.04	0.16	0.01
CeNi0.66Pd0.66	6.61	0.05	0.16	0.02
CeNi1Pd1	6.02	0.06	0.22	0.01
CeFe1Pd1	6.58	0.04	0.17	0.02
CeCo1Pd	5.34	0.06	0.19	0.01
Ce Ni0.89Pd1.1	2.84	0.04	0.25	0.01

**Table 4:** Estimated effective activation energy ($E_{\alpha }$) and kinetic rate for isothermal catalytic steam gasification of asphaltenes in the absence and presence with CeO_2_, CeNi1Pd1, and CeNi0.89Pd1.1 nanoparticles.

Sample	Temperature °C	$E_{\alpha }$ (kJ)	dα/dt Kinetic Rate (min^−1^) at 50% Conversion
*n*-C_7_ asphaltenes (without nanoparticles)	360	211.5	0.012
	370		0.018
	380		0.032
CeO_2_	230	44.0	0.013
	240		0.021
	250		0.879
CeNi1Pd1	230	29.6	0.0187
	240		0.0401
	250		0.1002
CeNi0.89Pd1.1	230		0.029
	240	25.6	0.084
	250		0.179

## Supplementary Materials

The following are available online at https://www.mdpi.com/2079-4991/9/3/401/s1, Figure S1. Raman spectra for the support (CeO_2_) and for the CeNi1Pd1 nanoparticles. For CeO_2_ a characteristic spectrum for fluorite was obtained, and for the NiPd-functionalized CeO_2_, a mixed of fluorite and spinel was obtained, confirming the presence of the Co_3_O_4_; Figure S2. Three components simplex-centroid mixture design with a ceria support (CeO_2_) functionalized with nickel oxide (NiO) and palladium oxide (PdO); Table S1: Calculated parameters of the Special Cubic Model for the *n*-C_7_ asphaltenes conversion time in the presence of SCMD nanoparticles.

## References

[1] Gill S., Tsolakis A., Dearn K., Rodríguez-Fernández J. (2011). Combustion characteristics and emissions of Fischer–Tropsch diesel fuels in IC engines. Prog. Energy Combust. Sci..

[2] Mitra-Kirtley S., Mullins O.C. (2007). Sulfur chemical moieties in carbonaceous materials. Asphaltenes, Heavy Oils, and Petroleomics.

[3] Lv Y., Tang D., Xu H., Luo H. (2012). Production characteristics and the key factors in high-rank coalbed methane fields: A case study on the Fanzhuang Block, Southern Qinshui Basin, China. Int. J. Coal Geol..

[4] Gray M.R., McCaffrey W.C. (2002). Role of chain reactions and olefin formation in cracking, hydroconversion, and coking of petroleum and bitumen fractions. Energy Fuels.

[5] Groenzin H., Mullins O.C. (2000). Molecular size and structure of asphaltenes from various sources. Energy Fuels.

[6] Calemma V., Iwanski P., Nali M., Scotti R., Montanari L. (1995). Structural characterization of asphaltenes of different origins. Energy Fuels.

[7] Piro G., Canonico L.B., Galbariggi G., Bertero L., Carniani C. (1996). Asphaltene adsorption onto formation rock: An approach to asphaltene formation damage prevention. SPE Prod. Facil..

[8] Yi S., Babadagli T., Li H.A. (2018). Use of nickel nanoparticles for promoting aquathermolysis reaction during cyclic steam stimulation. SPE J..

[9] Hashemi R., Nassar N.N., Pereira Almao P. (2013). In situ upgrading of Athabasca bitumen using multimetallic ultradispersed nanocatalysts in an oil sands packed-bed column: Part 1. Produced liquid quality enhancement. Energy Fuels.

[10] Sarathi P.S., Olsen D.K. (1992). Practical Aspects of Steam Injection Processes: A Handbook for Independent Operators.

[11] Barillas J., Júnior T.D., Mata W. (2008). Improved oil recovery process for heavy oil: A review. Braz. J. Petrol. Gás.

[12] Benavides Nieves L.D., Pinilla Najar L.A. (2017). Evaluación de la Viabilidad Técnica de la Inyección de vapor en Yacimientos de Crudo pesado, Mediante un Modelo Analítico.

[13] Nassar N.N., Franco C.A., Montoya T., Cortés F.B., Hassan A. (2015). Effect of oxide support on Ni–Pd bimetallic nanocatalysts for steam gasification of n-C7 asphaltenes. Fuel.

[14] Franco C., Cardona L., Lopera S., Mejía J., Cortés F. Heavy oil upgrading and enhanced recovery in a continuous steam injection process assisted by nanoparticulated catalysts. Proceedings of the SPE Improved oil Recovery Conference.

[15] Franco C.A. (2015). Synthesis and Application of Supported Metallic and Multi-Metallic Oxides Nanoparticles for In-Situ Upgrading and Inhibition of Formation Damage. Ph.D. Thesis.

[16] Nassar N.N., Hassan A., Pereira-Almao P. (2011). Application of nanotechnology for heavy oil upgrading: Catalytic steam gasification/cracking of asphaltenes. Energy Fuels.

[17] Franco C.A., Zabala R., Cortés F.B. (2017). Nanotechnology applied to the enhancement of oil and gas productivity and recovery of Colombian fields. J. Petrol. Sci. Eng..

[18] Guo K., Li H., Yu Z. (2016). In-situ heavy and extra-heavy oil recovery: A review. Fuel.

[19] Shah A., Fishwick R., Wood J., Leeke G., Rigby S., Greaves M. (2010). A review of novel techniques for heavy oil and bitumen extraction and upgrading. Energy Environ. Sci..

[20] Betancur S., Carmona J.C., Nassar N.N., Franco C.A., Cortés F.B. (2016). Role of particle size and surface acidity of silica gel nanoparticles in inhibition of formation damage by asphaltene in oil reservoirs. Ind. Eng. Chem. Res..

[21] Cortés F.B., Montoya T., Acevedo S., Nassar N.N., Franco C.A. (2016). Adsorption-desorption of n-c7 asphaltenes over micro-and nanoparticles of silica and its impact on wettability alteration. CT&F-Ciencia Tecnología y Futuro.

[22] López D., Giraldo L.J., Salazar J.P., Zapata D.M., Ortega D.C., Franco C.A., Cortés F.B. (2017). Metal Oxide Nanoparticles Supported on Macro-Mesoporous Aluminosilicates for Catalytic Steam Gasification of Heavy Oil Fractions for On-Site Upgrading. Catalysts.

[23] Nassar N.N., Betancur S., Acevedo S.c., Franco C.A., Cortés F.B. (2015). Development of a population balance model to describe the influence of shear and nanoparticles on the aggregation and fragmentation of asphaltene aggregates. Ind. Eng. Chem. Res..

[24] Adams J.J. (2014). Asphaltene adsorption, a literature review. Energy Fuels.

[25] Hamedi Shokrlu Y., Babadagli T. (2013). In-situ upgrading of heavy oil/bitumen during steam injection by use of metal nanoparticles: A study on in-situ catalysis and catalyst transportation. SPE Reserv. Eval. Eng..

[26] Nassar N.N., Hassan A., Pereira-Almao P. (2012). Thermogravimetric studies on catalytic effect of metal oxide nanoparticles on asphaltene pyrolysis under inert conditions. J. Therm. Anal. Calorim..

[27] Amanam U.U., Kovscek A.R. (2017). Analysis of the effects of copper nanoparticles on in-situ combustion of extra heavy-crude oil. J. Petrol. Sci. Eng..

[28] Nassar N.N., Hassan A., Luna G., Pereira-Almao P. (2013). Kinetics of the catalytic thermo-oxidation of asphaltenes at isothermal conditions on different metal oxide nanoparticle surfaces. Catal. Today.

[29] Franco C., Patiño E., Benjumea P., Ruiz M.A., Cortés F.B. (2013). Kinetic and thermodynamic equilibrium of asphaltenes sorption onto nanoparticles of nickel oxide supported on nanoparticulated alumina. Fuel.

[30] Cortés F.B., Mejía J.M., Ruiz M.A., Benjumea P., Riffel D.B. (2012). Sorption of asphaltenes onto nanoparticles of nickel oxide supported on nanoparticulated silica gel. Energy Fuels.

[31] Franco C.A., Montoya T., Nassar N.N., Pereira-Almao P., Cortés F.B. (2013). Adsorption and subsequent oxidation of colombian asphaltenes onto nickel and/or palladium oxide supported on fumed silica nanoparticles. Energy Fuels.

[32] Nassar N.N., Hassan A., Pereira-Almao P. (2011). Metal oxide nanoparticles for asphaltene adsorption and oxidation. Energy Fuels.

[33] Hashemi R., Nassar N.N., Pereira Almao P. (2013). Enhanced heavy oil recovery by in situ prepared ultradispersed multimetallic nanoparticles: A study of hot fluid flooding for Athabasca bitumen recovery. Energy Fuels.

[34] Nassar N.N., Hassan A., Pereira-Almao P. (2011). Comparative oxidation of adsorbed asphaltenes onto transition metal oxide nanoparticles. Colloids Surf. A Physicochem. Eng. Asp..

[35] Hashemi R., Nassar N.N., Almao P.P. (2014). Nanoparticle technology for heavy oil in-situ upgrading and recovery enhancement: Opportunities and challenges. Appl. Energy.

[36] Nassar N.N. (2010). Asphaltene adsorption onto alumina nanoparticles: Kinetics and thermodynamic studies. Energy Fuels.

[37] Cardona L., Arias-Madrid D., Cortés F.B., Lopera S.H., Franco C.A. (2018). Heavy Oil Upgrading and Enhanced Recovery in a Steam Injection Process Assisted by NiO-and PdO-Functionalized SiO_2_ Nanoparticulated Catalysts. Catalysts.

[38] Cardona Rojas L. (2018). Efecto de nanopartículas en procesos con inyección de vapor a diferentes calidades. Master’s Thesis.

[39] Jacobs G., Ricote S., Graham U.M., Patterson P.M., Davis B.H. (2005). Low temperature water gas shift: Type and loading of metal impacts forward decomposition of pseudo-stabilized formate over metal/ceria catalysts. Catal. Today.

[40] Brunauer S., Emmett P.H., Teller E. (1938). Adsorption of gases in multimolecular layers. J. Am. Chem. Soc..

[41] Dejhosseini M., Aida T., Watanabe M., Takami S., Hojo D., Aoki N., Arita T., Kishita A., Adschiri T. (2013). Catalytic cracking reaction of heavy oil in the presence of cerium oxide nanoparticles in supercritical water. Energy Fuels.

[42] Nassar N.N., Hassan A., Vitale G. (2014). Comparing kinetics and mechanism of adsorption and thermo-oxidative decomposition of Athabasca asphaltenes onto TiO_2_, ZrO_2_, and CeO_2_ nanoparticles. Appl. Catal. A Gen..

[43] Sánchez Gil J.J. (2013). Síntesis y Estudios de Catalizadores Nanoestructurados de Óxido de Cerio soportado sobre Óxido de Magnesio con baja cantidad en Lantánido. Master’s Thesis.

[44] Li Y., Fu Q., Flytzani-Stephanopoulos M. (2000). Low-temperature water-gas shift reaction over Cu-and Ni-loaded cerium oxide catalysts. Appl. Catal. B Environ..

[45] Bunluesin T., Gorte R., Graham G. (1998). Studies of the water-gas-shift reaction on ceria-supported Pt, Pd, and Rh: Implications for oxygen-storage properties. Appl. Catal. B Environ..

[46] Meunier F., Reid D., Goguet A., Shekhtman S., Hardacre C., Burch R., Deng W., Flytzani-Stephanopoulos M. (2007). Quantitative analysis of the reactivity of formate species seen by DRIFTS over a Au/Ce (La) O_2_ water–gas shift catalyst: First unambiguous evidence of the minority role of formates as reaction intermediates. J. Catal..

[47] Alamolhoda S., Vitale G., Hassan A., Nassar N.N., Almao P.P. (2019). Synergetic effects of cerium and nickel in Ce-Ni-MFI catalysts on low-temperature water-gas shift reaction. Fuel.

[48] Ancheyta J., Centeno G., Trejo F., Marroquin G., Garcia J., Tenorio E., Torres A. (2002). Extraction and characterization of asphaltenes from different crude oils and solvents. Energy Fuels.

[49] Franco C.A., Nassar N.N., Montoya T., Ruíz M.A., Cortés F.B. (2015). Influence of asphaltene aggregation on the adsorption and catalytic behavior of nanoparticles. Energy Fuels.

[50] Choi S., Byun D.H., Lee K., Kim J.-D., Nho N.S. (2016). Asphaltene precipitation with partially oxidized asphaltene from water/heavy crude oil emulsion. J. Petrol. Sci. Eng..

[51] Lozano M.M., Franco C.A., Acevedo S.A., Nassar N.N., Cortés F.B. (2016). Effects of resin I on the catalytic oxidation of n-C 7 asphaltenes in the presence of silica-based nanoparticles. RSC Adv..

[52] Franco C.A., Zabala R.D., Zapata J., Mora E., Botero O., Candela C., Castillo A. (2013). Inhibited gas stimulation to mitigate condensate banking and maximize recovery in cupiagua field. SPE Prod. Oper..

[53] Chen R., Zhang Z., Feng C., Hu K., Li M., Li Y., Shimizu K., Chen N., Sugiura N. (2010). Application of simplex-centroid mixture design in developing and optimizing ceramic adsorbent for As (V) removal from water solution. Microporous Mesoporous Mater..

[54] Talu O., Meunier F. (1996). Adsorption of associating molecules in micropores and application to water on carbon. AIChE J..

[55] Montoya T., Coral D., Franco C.A., Nassar N.N., Cortés F.B. (2014). A Novel Solid–Liquid Equilibrium Model for Describing the Adsorption of Associating Asphaltene Molecules onto Solid Surfaces Based on the “Chemical Theory”. Energy Fuels.

[56] Cao A., Lu R., Veser G. (2010). Stabilizing metal nanoparticles for heterogeneous catalysis. Phys. Chem. Chem. Phys..

[57] Allen K.M., Auyeung N., Rahmatian N., Klausner J.F., Coker E.N. (2013). Cobalt ferrite in YSZ for use as reactive material in solar thermochemical water and carbon dioxide splitting, part II: Kinetic modeling. JOM.

[58] Sharma A., Saito I., Nakagawa H., Miura K. (2007). Effect of carbonization temperature on the nickel crystallite size of a Ni/C catalyst for catalytic hydrothermal gasification of organic compounds. Fuel.

[59] Stone H. (1968). Electrical conductivity and sintering in iron oxides at high temperatures. J. Mater. Sci..

[60] Phalnikar C., Evans E., Baldwin W. (1956). High Temperature Scaling of Cobalt-Chromium Alloys. J. Electrochem. Soc..

[61] Ravikovitch P.I., Neimark A.V. (2001). Characterization of nanoporous materials from adsorption and desorption isotherms. Colloids Surf. A Physicochem. Eng. Asp..

[62] Franco C.A., Montoya T., Nassar N.N., Cortés F.B. (2014). Nioand pdo supported on fumed silica nanoparticles for adsorption and catalytic steam gasification of colombian c7asphaltenes. Handbook on Oil Production Research.

[63] Headen T., Boek E., Jackson G., Totton T., Muller E. (2017). Simulation of asphaltene aggregation through molecular dynamics: Insights and limitations. Energy Fuels.

[64] Bates M.K., Jia Q., Doan H., Liang W., Mukerjee S. (2015). Charge-transfer effects in Ni–Fe and Ni–Fe–Co mixed-metal oxides for the alkaline oxygen evolution reaction. ACS Catal..

[65] Shido T., Iwasawa Y. (1993). Reactant-promoted reaction mechanism for water-gas shift reaction on Rh-doped CeO_2_. J. Catal..

[66] Vignatti C.I., Avila M.S., Apesteguia C.R., Garetto T.F. (2011). Study of the water-gas shift reaction over Pt supported on CeO_2_–ZrO_2_ mixed oxides. Catal. Today.

