# Supplementary material for: Optimization of the Load of Transition Metal Oxides (Fe2O3, Co3O4, NiO and/or PdO) onto CeO2 Nanoparticles in Catalytic Steam Decomposition of n-C7 Asphaltenes at Low Temperatures
Source: Nanomaterials (Basel). 2019 Mar 9;9(3):401. doi: 10.3390/nano9030401 (PMC6474133; doi:10.3390/nano9030401)
Supplement: Supplementary file 1 [file nanomaterials-09-00401-s001.pdf]

nanomaterials

Supplementary material for

Optimization of the Load of Transition Metal Oxides (Fe_2O_3, Co_3O_4, NiO and/or PdO) onto CeO_2 Nanoparticles in Catalytic Steam Decomposition of *n*-C_7 Asphaltenes at Low Temperatures

Oscar E. Medina[1], Jaime Gallego[2], Daniela Arias-Madrid[1], Farid B. Cortés[1,*] and Camilo A. Franco[1,*]

[1] Grupo de Investigación en Fenómenos de Superficie—Michael Polanyi, Departamento de Procesos y Energía, Facultad de Minas, Universidad Nacional de Colombia, Sede Medellín, 050034 Medellín, Colombia; oemedinae@unal.edu.co (O.E.M.) , daariasma@unal.edu.co (D.A.M.)

[2] Química de Recursos Energéticos y Medio Ambiente, Instituto de Química, Universidad de Antioquia UdeA, Calle 70 No. 52-21, Medellín, Colombia; andres.gallego@udea.edu.co (J.G.)

* Correspondence: caafrancoar@unal.edu.co (C.A.F.) and fbcortes@unal.edu.co (F.B.C.)

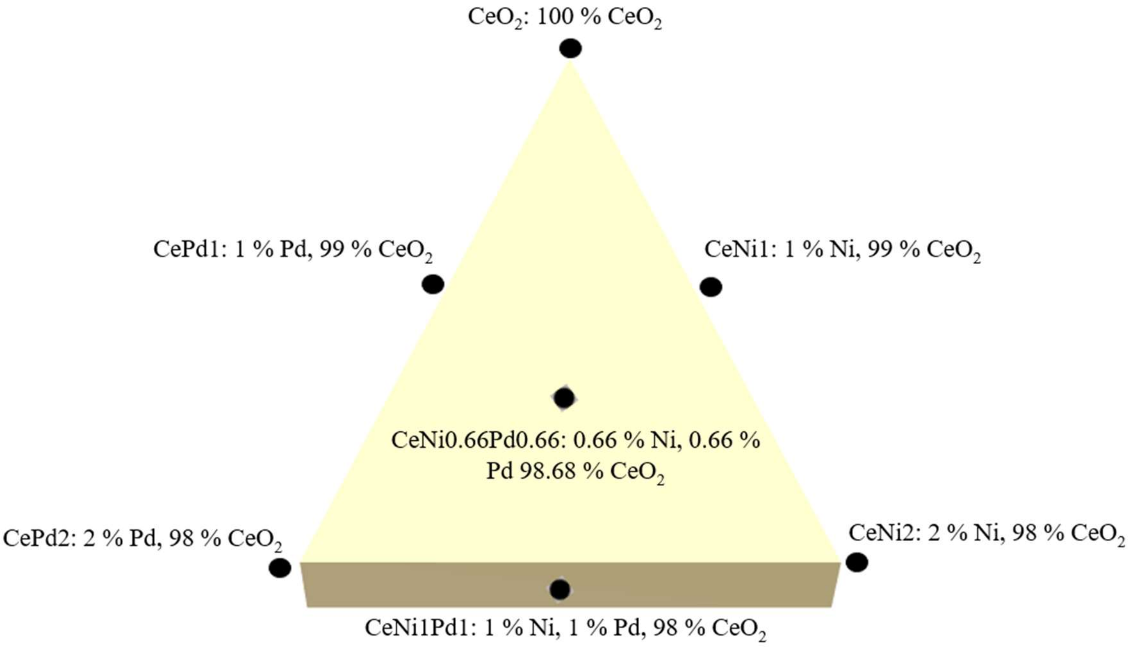

Figure S1. Three components simplex-centroid mixture design with a ceria support (CeO_2) functionalized with nickel oxide (NiO) and palladium oxide (PdO).

Table S1. Calculated parameters of the Special Cubic Model for the n-C_7 asphaltenes conversion time in the presence of SCMD nanoparticles.

β_1	β_2	β_3	β_{12}	β_{13}	β_{23}	β_{123}	R^2
0.006896	0.008264	0.008928	0.000246	0.002873	0.006017	0.003310	0.99

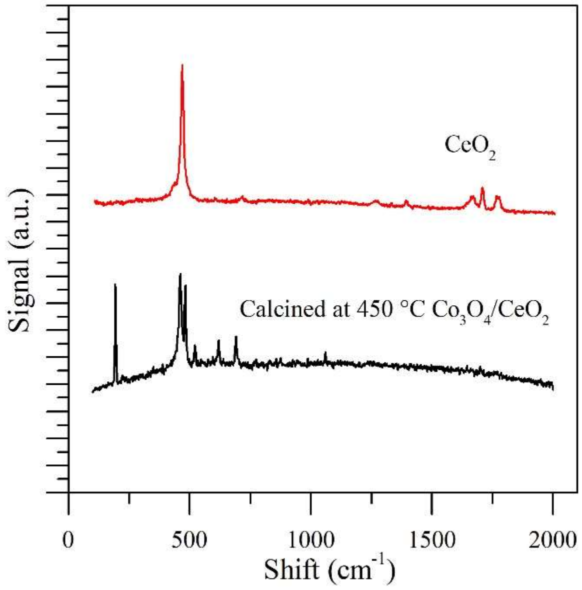

Figure S2. Raman spectra for the support (CeO_2) and for the NiPd/CeO_2 nanoparticles. For CeO_2 a characteristic spectrum for fluorite was obtained, and for the NiPd functionalizated CeO_2, a mixed of fluorite and spinel was obtained, confirming the presence of the Co_3O_4.
